# Non-suicidal self-injury maintenance and cessation among adolescents: a one-year longitudinal investigation of the role of objectified body consciousness, depression and emotion dysregulation

**DOI:** 10.1186/s13034-015-0052-9

**Published:** 2015-07-08

**Authors:** Jamie Duggan, Nancy Heath, Tina Hu

**Affiliations:** Department of Educational & Counselling Psychology, McGill University, Montreal, QC Canada; Faculty of Medicine, University of Toronto, Toronto, ON Canada

## Abstract

Using the objectification theory, scholars have theorized the sense of detachment and disregard for the body that results from continued body objectification are believed to put a person at greater risk for non-suicidal self-injury (NSSI), due to a lack of emotional investment in the body. The goal of the current study was to longitudinally investigate the association between body objectification and NSSI among an early adolescent sample. The overall sample consisted of 120 participants (56 % female) who ranged in age from 11 to 13 years of age (*M* = 12.34, *SD* = .48). Participants were followed over the course of a 12-month period, and classified into three groups of interest; adolescents who reported maintaining NSSI behaviour over the course of a year (NSSI Maintain group, *n* = 20), adolescents who reported stopping the behaviour over the course of a year (NSSI Stop group, *n* = 40), and a comparison group of adolescents who did not report engaging in NSSI (*n* = 60). Using a 3 (NSSI Maintain, NSSI Stop, and Comparison) X 2 (Gender) X 2 (Time 1 and Time 2) repeated measures multiple analysis of variance (MANOVA), results indicated a significant group by time interaction, showing group differences with respect to body shame and body surveillance over time. Specifically, both NSSI groups reported significantly greater body shame and body surveillance over time than the non-NSSI group. Additionally, the NSSI Maintain group reported significantly greater body surveillance at T2 when compared to the NSSI Stop and non-NSSI group. The NSSI Maintain group also reported significantly more emotion dysregulation difficulties and depressive symptoms at T2 when compared to the NSSI Stop and non-NSSI group. The influence of body objectification as a core intrapersonal risk factor related to the maintenance and cessation of NSSI behaviour is discussed, as are clinical implications considering body objectification as an important variable in prevention and treatment efforts.

In recent years, the study of non-suicidal self-injury (NSSI) among community populations has grown considerably. Defined as the intentional, self-inflicted destruction of body tissue resulting in immediate damage, which is done without suicidal intent and for purposes not culturally sanctioned [1], prevalence rates range from 38 % to 82 % among clinical adolescent populations [2, 3], and 21 % to 65 % among adults [4, 5]. Community prevalence rates are comparable, and range from 12 % to 20 % among young adults [6, 7] and 14 % to 26 % among adolescents [8–10]. The developmental period of adolescence appears to represent a time of particular risk for NSSI engagement. In addition to significantly higher prevalence rates, the majority of youth who report engaging in NSSI describe the behaviour as beginning between the ages of 12 and 15 years [11, 12]. Despite reports that many adolescents typically stop engaging in NSSI within five years of the initial onset, the behaviour can often persist into adulthood [7]. Thus, it appears that adolescence represents a developmental period that is associated with the emergence, maintenance, and to some degree, the cessation of the behaviour.

Emotion regulation models have received significant support within the literature, conceptualizing NSSI as a behaviour that is motivated and maintained by its emotion regulation properties [13–15]. These models have received substantial empirical support among clinical and non-clinical samples of adolescents and young adults [13, 16, 17]. Although emotion regulation difficulties are necessary precursors, they do not sufficiently explain factors that may predispose an individual to choose NSSI, nor the mechanisms supporting one’s decision to use the behaviour as opposed to other less physically harmful coping strategies. Therefore, additional risk factors merit inclusion into etiological models of NSSI. The body-oriented behaviours that characterize NSSI have led scholars to examine the way in which those who self-injure experience the body as a valuable intrapersonal factor worthy of investigation.

Within the literature, researchers have recognized that a negative view of the body represents a critical risk factor related to NSSI [9, 18–24]. As argued by Orbach [22], negative views and attitudes regarding body experiences (e.g., rejection of the bodily self) reduce the likelihood of self-preservation, leading to a reduction in an individual’s “natural shield protecting the body,” and facilitating the decision to engage in self-destructive behaviours [22]. Related to Orbach’s framework, objectification theory [25] represents a theory rooted in feminism, which argues that women internalize societal objectification of the female body, learning to habitually self-monitor their body, which contributes to the view that one’s own body is an object to be evaluated from an outsider’s perspective [25]. Continued body objectification results in the development of an objectified body consciousness, a concept proposed by McKinley and colleagues, which includes elevated levels of body shame, and continual monitoring of one’s appearance [26, 27]. Using objectification theory as a theoretical lens, scholars argued that the sense of detachment and disregard for the body that results from body objectification is believed to put a person at greater risk for NSSI, due to reduced emotional investment in the body [22, 24].

A small but growing body of cross-sectional studies supports an association between adolescents who report engaging in NSSI and negative views of the body. Specifically, Ross and colleagues [23] examined the role of eating pathology, body image, general self-concept, gender, and NSSI among 440 high school adolescents. Results demonstrated that high school students with a history of NSSI reported a greater body focused orientation, which included being more dissatisfied with the shape and size of their body, as well as greater feelings of inadequacy, insecurity, and worthlessness in comparison to their non-NSSI peers [23]. Building upon these findings, Brausch and Gutierrez [28] examined differences among 373 adolescents (48 % female) reporting varying levels of self-harming behaviours (i.e., no history of self-harming behaviours, NSSI only, and NSSI with a history of suicidality). The authors reported that body dissatisfaction was significantly higher and self-esteem was significantly lower in both of the NSSI groups when compared to the comparison group, but these factors did not distinguish the two NSSI groups. These findings illustrate that youth who report engaging in any degree of self-harming behaviours view themselves and their bodies differently than youth who do not.

Extending upon this line of inquiry, Muehlekamp and Brausch [29] investigated the association between body image and NSSI and included a measure of negative affect. The authors used structural equation modeling to evaluate an etiological model of NSSI risk that proposed that negative body image mediated the relationship between negative affect (i.e., depression and hopelessness) and NSSI among a combined clinical (various diagnoses, including oppositional defiant disorder, conduct disorder, and major depressive disorder) and non-clinical sample of 284 adolescents (75 % female). Results indicated that the model accounted for 22 % of the variance in NSSI, with negative body image serving as a significant mediator. Based on these findings, the authors concluded that negative body image represents a necessary, but not sufficient risk factor related to NSSI engagement. Thus, adolescents who maintained a negative view of the body were more likely to engage in NSSI when confronted with overwhelming emotional distress. Expanding on current findings to include the role of gender, Nelson and Muehlenkamp [18] investigated gender differences in body objectification (i.e., body surveillance and body shame), body image, and body esteem among 251 young adults (82 % female) with and without a history of NSSI. The researchers reported that individuals with a history of NSSI reported higher self-objectification, higher body shame, and lower levels of body esteem.

## Rationale

To date, a growing body of evidence demonstrates that negative body image and experiences are more prevalent among adolescents who engage in NSSI when compared to their non-NSSI peers. Emphasizing the role of the body as a risk factor related to NSSI may help to partially explain why the developmental period of adolescence represents such a crucial time associated with high prevalence rates and emergence of the behaviour [11, 12]. To begin, the onset of adolescence is characterized by numerous physical and emotional changes (i.e., pubertal onset, emerging sexuality, identify formation, and gender role intensifications), and it is also when the ability to cognitively engage in body objectification typically begins to emerge [30, 31]. Consequently, it has been reported that both adolescent girls and boys report significant concerns over their bodies during this time [30, 32, 33]. This period represents a critical time to examine the association between objectified body consciousness and NSSI in order to gain an accurate developmental understanding of how negative views of the body facilitate NSSI engagement.

A main limitation within the literature concerns the cross-sectional nature of the majority of studies. Researchers have not yet determined if there is in fact a direct causal relationship between the unstable nature of body image during adolescence and the emergence, maintenance, and cessation of NSSI. Longitudinal research would assist in clarifying the directional nature of the relationships between these variables. A secondary limitation within the literature concerns the investigation of the role of gender. With the exception of a few studies, the bulk of the literature has primarily focused on examining this relationship among females. Very few studies have examined the association between objectified body consciousness, depression, and emotion dysregulation among an adolescent sample of males and females over time. Doing so would validate the role of objectification theory by demonstrating that it is a core risk factor associated with NSSI. Thus, when taken together, there is a need for studies to longitudinally investigate the development of objectified body consciousness, depression, emotion dysregulation, and NSSI during early adolescence, in order to develop an accurate developmental understanding of the objectification pathway.

### Research objectives

The purpose of the current study was to expand upon research on the role of the body in NSSI engagement, by examining temporal changes in body-objectification, depression, emotion dysregulation, and gender, among three groups of interest. Specifically, among adolescents who reported maintaining NSSI behaviour over the course of a year (NSSI Maintain group), adolescents who reported stopping the behaviour over the course of a year (NSSI Stop group), and a comparison group of adolescents who did not report engaging in NSSI or risky behaviours (e.g., health risk behaviours which included drug and/or alcohol use, smoking, overeating, physical fighting) over the course of a year. In summary, there were two main goals for the present study. The first research objective was to investigate group and gender differences across three dimensions of body objectification (i.e., body shame, body surveillance, appearance control beliefs) over time. The second objective was to examine changes in depression and emotion dysregulation across groups and gender.

## Method

### Participants

The current study represents a subset of data collected over 2 years as part of a larger three-year longitudinal project investigating stress and coping strategies among 906 grade 7 students. Participants were recruited from 15 high schools in Montreal, Quebec. The overall sample consisted of 501 female (55 %) and 392 male (44 %) participants, with 13 participants missing gender data (1 %). Participants ranged in age from 11 to 13 years of age (*M* = 12.34, *SD* = .48) and reported their place of birth as Canada (96 %), followed by the United States (2 %), and other countries (3 %).

From the overall sample in grade 7, 7 % of participants (*n* = 66) indicated that they had engaged in NSSI at least once in their lifetime on a screening measure and were classified into the NSSI group. This group consisted of 37 female participants (56 %) and 29 male participants (44 %). A total of 15 % (*n* = 10) reported having engaged in NSSI only once, 29 % (*n* = 19) reported 2 to 4 times, 18 % (*n* = 12) reported 5 to 10 times, 18 % (*n* = 12) reported 11 to 50 times, 6 % (*n* = 4) reported 51 to 100 times, and 6 % (*n* = 4) of participants reported having engaged in NSSI 100 or more times. From the participants who self-injured, 52 % reported having engaged in the behaviour within the last three months.

Of the 906 participants who participated at T1, 825 (91 %) completed the assessments 12 months later (i.e., T2), when students were in grade eight. Attrition was due to incompletion of the questionnaires (*n* = 6), withdrawal (*n* = 27), absenteeism (*n* = 10), and moving to a different school (*n* = 38). From the overall sample, 20 participants reported engaging in NSSI at T1 and then again at T2 (70 % female). These participants were classified into the NSSI Maintain group. An additional 40 participants (50 % female) reported engaging in NSSI at T1, but no longer reported engaging in the behaviour at T2. These participants were classified into the NSSI Stop group. A comparison group of adolescents (*n* = 60; 57 % female) who did not report engaging in NSSI during T1 or T2 was created by matching participants on gender and school from the same pool of students through random number generation.

### Measures

All measures were administered at T1 and then 12-months later at T2.

### How I deal with stress questionnaire (HIDS) [34]

The HIDS was originally developed and reported by Ross and Heath [12] and is a 31-item self-report questionnaire that presents a list of strategies derived through a review of the literature concerning adaptive and maladaptive coping strategies used to manage stress and other difficulties. The HIDS was used to collect demographic information and served to screen for the presence or absence of NSSI behaviour. The first section of the HIDS collected demographic information from participants, which included age, gender, sexual orientation, languages spoken within the home, country of permanent residence, and country of birth. In the second section, participants were asked to rate their use of 31 adaptive (e.g., read, exercise) and maladaptive (e.g., drink, stop eating, physically hurt myself on purpose) coping strategies for stress on a four-point Likert scale (0 = never; 3 = always). Adolescents who indicated that they have ever physically hurt themselves on purpose in their lifetime as a way to cope with stress were prompted to complete a follow-up section, where they had to indicate which behaviours they have used to intentionally hurt themselves without suicidal intent (e.g., cutting, hitting). They also had to report on feelings experienced after having engaged in NSSI behaviour, lifetime, and three-month prevalence rates of their reported self-injury, and whether or not they have stopped engaging in the behaviour.

The HIDS questionnaire section examining the use of adaptive and maladaptive coping strategies for stress was found to have a high degree of internal consistency (31 items; α = .80). This value is consistent with psychometric analyses conducted in previous studies utilizing the HIDS, which suggests that the items on the questionnaire form a scale with reasonable internal consistency [6]. Test-retest reliability of the NSSI screening item (i.e., “physically hurt myself on purpose”) is reported as high (*r* = .83) [35].

### Body objectification

The Objectified Body Consciousness Scale-Youth (OBCS-Y) [36] is a 14-item self-report measure, which comprises three subscales designed to assess three aspects of objectified body consciousness. The body surveillance subscale consists of items measuring body objectification in the form of appearance monitoring and adopting an outsider’s view of the self (i.e., “I often worry about how I look to other people”). The body shame subscale measures feelings of inadequacy and shame surrounding one’s view of their body (i.e., “I feel ashamed of myself because of my physical appearance”). The appearance control subscale measures perceived control over physical appearance (i.e., “I think I could look as good as I wanted to if I worked at it”). Items are answered according to a seven-point scale, ranging from “strongly agree” to “strongly disagree”. Scores are obtained by averaging item responses within each subscale, with higher scores indicating greater degrees of surveillance, body-shame, and body surveillance. The OBCS-Y has demonstrated adequate two-week test-retest reliability for all three subscales (*r* = .81 for surveillance, *r* = .62 for body shame, and *r* = .70 for control beliefs). Validity is supported by moderate to significant correlations with other measures of body esteem, dissatisfaction, and appearance orientation [36].

### Depressive symptoms

To assess for depressive symptoms, the Beck Depression Inventory (BDI) of the Beck Youth Inventories – Second Edition (BYI-II) [37] was used. The BDI-Y is a self-report inventory that contains 20 statements about thoughts, feelings, and behaviours in youth aged 7 to 18 years. The BDI-Y includes items related to an adolescent’s negative thoughts about self, life, and the future, feelings of sadness and guilt, and sleep disturbance. The BDI-Y has demonstrated high internal consistency and validity is supported by moderate to significant correlations on self-report measures of hopelessness, anxiety, and suicide-related behaviours [38].

### Emotion dysregulation

Emotion regulation difficulties were assessed using three questions from the Difficulty in Emotion Regulation Scale (DERS) [39], a 36-item inventory designed to assess difficulties in emotion regulation. The three questions used in this study assessed the dimensions of (a) Difficulties Engaging in Goal Directed Behaviour (i.e., “When I’m upset, I have difficulty thinking about anything else”), (b) Impulse Control Difficulties (i.e., “When I’m upset, I feel out of control”), and (c) Limited Access to Emotion Regulation Strategies (i.e., “When I’m upset, there’s nothing I can do to make myself feel better”). The selected questions were chosen as they were the most indicative of emotion regulation difficulties among individuals who engage in NSSI [40]. The inventory asks participants to indicate how often each statement is true for them, using a five-point Likert scale, ranging from “almost never” to “almost always”. The DERS questions had high internal consistency (three items; α = .72).

### Procedure

Following review and ethics board approval for the longitudinal project, all grade 7 students from 15 participating schools were invited to participate in the study. Data collection began with a presentation to all eligible students at participating schools in order to obtain informed consent. This presentation described the study as a project examining stress and coping strategies among high school students and outlined the goals and objectives of the project, methodology, what is required of participants (e.g., time commitment and questionnaires students will be asked to complete), and the benefits of participating. Students were provided with a project information letter and consent form and had the opportunity to ask questions and express any concerns at that time. Informed consent was then sought from parents. Parents were provided with a letter describing the project, the activities that their child would participate in, as well as the benefits of participation. These project information letters, along with an attached consent form, were sent home with all students (*N* = 2675). Of these students, 1312 students returned consent forms (49 %). Of these 1312 students, 906 students indicated an agreement to participate (68 %).

While students were encouraged to participate, they were informed that they had the option to withdraw from the study at any time. It was also emphasized that their participation would have no bearing on any class grades or evaluation. Students who had parental consent to participate in the study completed an assent form, which provided detailed information about the main research purposes, procedure, and compensation. Students were informed that they would receive compensation (entered in a draw for one of two gift cards to a local shopping mall in the amounts of $200 and $100) for returning the consent form, regardless of agreement to participate, as well as compensation for completing the Standard Assessment Battery (SAB) session (entered in a draw for one of four gift cards to a local movie theater in the amount of $50).

Following the obtainment of consent and assent, participants were administered the SAB, which consisted of the HIDS, BDI-Y, DERS items, and OBCS-Y, among other measures (which were part of the larger study). The SAB sessions were conducted with approximately 20 students (chosen from the participant pool) and took approximately 60 min to complete. These sessions occurred during school hours in a classroom that was allocated to the research team. The SAB session provided information regarding NSSI group classification. A follow-up individual interview was then completed with participants who completed the screening and met criteria for the NSSI group. Only once the participant confirmed their NSSI status during the individual interview were they officially included into the study. Graduate students in school psychology trained by a psychologist regarding suicide risk assessments conducted all interviews. Confidentiality was broken only in the event that the participant indicated to the interviewer that they were at risk of harming themselves or others. Once confidentiality was broken, the participant was informed and transitioned to a pre-determined school mental health professional (i.e., school psychologist, school counsellor) that was aware of the nature of the project as agreed in the research ethics board approval.

All schools involved in the research project were then contacted again in the fall of the following academic year to schedule dates for the second collection of data (T2), approximately 12 months from when the first data collection occurred. The same procedure and measures were followed at T2.

## Results

Prior to conducting analyses, all variables were examined through SPSS for accuracy of data entry, missing values, fit between their distributions, and assumptions of multivariate analyses. Of the 906 participants who participated at T1, 825 (91 %) completed the assessments 12 months later (i.e., T2), when students were in grade eight. In T1, 66 participants reported engaging in NSSI behaviour. Of these 66 participants, 20 participants reported engaging in NSSI at T2 (70 % female). These participants were classified into the NSSI Maintain group. An additional 40 participants (50 % female) reported stopping NSSI behaviour at T2. These participants were classified into the NSSI Stop group. Six participants were removed from the analysis due to ambiguous or missing responses that did not allow for clear group classification. A comparison group of adolescents (*n* = 60; 57 % female) who did not report engaging in NSSI was created by matching participants on gender and school from the same pool of students through random number generation. Refer to Table 1 for descriptive statistics of all measures in T1 and Table 2 for descriptive statistics of all measures in T2.

**Table 1:** Means and Standard Deviations for OBCS Subscales, DERS, and BDI by Group and Gender During Time 1

	Time 1					
	NSSI Maintain		NSSI Stop		Comparison	
	Males (*n* = 6)	Females (*n* = 14)	Males (*n* = 20)	Females (*n* = 20)	Males (*n* = 26)	Females (*n* = 34)
	*M (SD)*	*M (SD)*	*M (SD)*	*M (SD)*	*M (SD)*	*M (SD)*
OBCS Subscales						
Body Control	5.44 (0.65)	4.41 (1.16)	4.57 (0.96)	4.23 (0.71)	5.04 (0.95)	4.64 (0.98)
Body Shame	3.36 (1.02)	3.92 (1.19)	3.19 (1.05)	3.77 (1.05)	2.40 (0.59)	2.90 (0.99)
Body Surveillance	3.98 (0.43)	5.24 (0.80)	4.09 (1.24)	4.55 (0.85)	3.39 (0.96)	3.64 (1.24)
DERS	5.17 (2.56)	5.68 (2.11)	4.11 (2.97)	4.17 (2.46)	2.35 (2.12)	3.26 (2.40)
BDI	23.50 (14.98)	24.14 (12.84)	17.67 (13.43)	19.61 (12.78)	4.85 (4.86)	7.76 (6.15)

**Table 2:** Means and Standard Deviations for OBCS Subscales, DERS, and BDI by Group and Gender During Time 2

	Time 2					
	NSSI Maintain		NSSI Stop		Comparison	
	Males (*n* = 6)	Females (*n* = 14)	Males (*n* = 20)	Females (*n* = 20)	Males (*n* = 26)	Females (*n* = 34)
	*M (SD)*	*M (SD)*	*M (SD)*	*M (SD)*	*M (SD)*	*M (SD)*
OBCS Subscales						
Body Control	3.12 (0.98)	3.47 (1.26)	2.70 (0.94)	3.84 (0.89)	2.57 (0.76)	3.02 (0.97)
Body Shame	5.15 (1.29)	4.72 (0.99)	4.96 (1.08)	4.44 (0.81)	5.03 (1.03)	4.52 (0.73)
Body Surveillance	4.13 (0.71)	5.36 (0.93)	3.69 (1.20)	4.62 (0.84)	3.53 (1.09)	4.16 (1.09)
DERS	5.83 (3.60)	3.43 (2.59)	2.28 (2.16)	4.11 (2.65)	1.77 (1.58)	2.88 (1.81)
BDI	20.50 (6.54)	24.63 (8.18)	11.72 (11.58)	17.50 (7.57)	6.81 (6.29)	8.56 (6.95)

### Relationship between NSSI and body objectification

To investigate group and gender differences in body objectification over time, a 3 (NSSI Maintain, NSSI Stop, and Comparison) X 2 (Gender) X 2 (Time 1 and Time 2) repeated measures multiple analysis of variance (MANOVA) was conducted. The dependent variables were the individual OBCS subscales (i.e., body shame, body surveillance, and appearance control beliefs), with group and gender as the independent variables. Descriptive statistics for all measures by gender across groups in T1 and T2 are listed in Table 1 and Table 2, respectively.

The interaction between group and time was significant, Wilks’ λ = .86, *F*(6, 224) = 2.89, *p* = .01, ηp^2^ = .07, indicating that groups differed with respect to body objectification as a function of time. An examination of the univariate within-group effects indicated significant group differences across time on two subscales; body shame and appearance control beliefs. Specifically, at T1, the NSSI Maintain (*M =*3.75; *SD =* 1.14) and NSSI Stop group (*M =*3.47; *SD =* 1.07) reported significantly more body shame when compared to the comparison group (*M =*2.68; *SD =* 0.87). Over time, all three groups reported an increase in body shame, however, at T2, significant differences were no longer observed between the NSSI Stop group (*M =*4.70; *SD =* .97) and the comparison group (*M =*4.74; *SD =* 0.90). Furthermore, all three groups reported a decrease in appearance control beliefs over time. Specially, at T1, the comparison group reported significantly more appearance control beliefs (*M =*4.81; *SD =* .97) than both the NSSI Maintain group (*M =*4.71; *SD =* 1.12) and the NSSI Stop group (*M =*4.39; *SD =* .85). However at T2, the comparison group reported significantly less control beliefs (*M =*2.82; *SD =* .90) than both the NSSI Maintain group (*M =*3.36; *SD =* 1.16) and NSSI Stop group (*M =*3.27, *SD =* 1.07).

A Hochberg’s GT2 post-hoc analysis was conducted for between-group comparisons at T2. This analysis was chosen due to its recommended use for unequal samples sizes [41]. Results demonstrated that at T2, the NSSI Maintain group (*M =*4.99; *SD =* 1.03) reported significantly more body surveillance than both the NSSI Stop group (*M =*4.15; *SD =* 1.12) and the comparison group (*M =*3.88; *SD =* 1.12). Differences were not observed between the NSSI Stop and comparison group at T2 with respect to body surveillance.

**Fig. 1:**
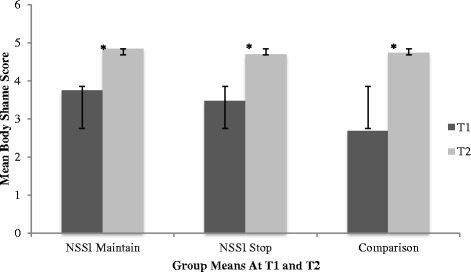
Bar chart representing group differences in OBCS Body Shame subscale over time*.* * = significant differences*.* Error bars denote one standard deviation around the mean

**Fig. 2:**
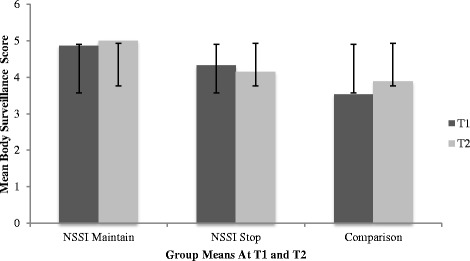
Bar chart representing group differences in OBCS Body Surveillance subscale over time*.* * = significant differences. Error bars denote one standard deviation around the mean

**Fig. 3:**
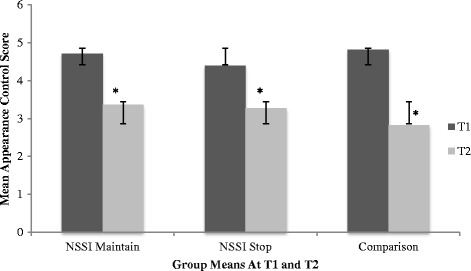
Bar chart representing group differences in OBCS Appearance Control subscale over time*.* * = significant differences. Error bars denote one standard deviation around the mean

The interaction between time and gender was also significant, Wilks’ λ = .83, *F*(3, 112) = 7.58, *p* < .00, ηp^2^ = .17. Univariate effects indicated males and females scored differently with respect to appearance control beliefs and body shame over time, regardless of group status. Specifically, both males and females reported increased body shame over time, regardless of group status. Females indicated increased body surveillance over time while males indicated decreased body surveillance over time, regardless of group status. Further, males and females reported decreased appearance control beliefs over time, regardless of group status. The triple interaction between time, group, and gender was not significant, Wilks’ λ = .98, *F*(6, 224) = .48, *p =* n.s., ηp^2^ = .01, indicating that body objectification did not differ over time between groups as a function of gender. Descriptive statistics by gender across groups in T1 and T2 are listed in Table 1 and Table 2, respectively. Refer to Table 3 for a detailed presentation of the univariate effects of group and gender on OBCS subscales across time. See Figs. 1, 2, and 3 for group mean differences with respect to OBSC subscales across time.

**Table 3:** Univariate effects of group and gender on OBCS subscales across time

Dependent Variables	*df*	*F*	ηp^2^	Observed Power	*p*
Group Membership					
Body Control	2	1.33	.02	.28	.27
Body Shame	2	6.95	.11	.92	.00**
Body Surveillance	2	10.12	.15	.98	.00**
Gender					
Body Control	1	0.04	.00	.05	.85
Body Shame	1	0.05	.00	.06	.83
Body Surveillance	1	17.67	.13	.99	.00**
Time					
Body Control	1	124.31	.52	1.00	.00**
Body Shame	1	110.99	.49	1.00	.00**
Body Surveillance	1	1.69	.01	.13	.41
Group X Time					
Body Control	2	5.46	.09	.84	.01*
Body Shame	2	5.81	.09	.86	.00*
Body Surveillance	2	2.39	.04	.48	.10
Time X Gender					
Body Control	1	18.51	.14	.99	.00**
Body Shame	1	12.30	.10	.94	.00**
Body Surveillance	1	1.39	.01	.22	.24
Time X Group X Gender					
Body Control	2	0.74	.01	.17	.48
Body Shame	2	0.02	.00	.05	.99
Body Surveillance	2	0.30	.01	.10	.74

* = *p* < .01; ** = *p* < .001

### Depression and emotion dysregulation

To investigate group and gender differences in depression and emotion dysregulation over time, a three (NSSI Maintain, NSSI Stop, Comparison) X 2 (Gender) X 2 (Time 1 and Time 2) repeated measures analysis of variance (ANOVA) was conducted. The dependent variables were the total score of the three DERS items and the BDI-Y total score. Results obtained from the repeated measures ANOVA showed that the main effect for time was significant, Wilks’ λ = .94*, F*(2, 109) = 3.46, *p =* .04, ηp^2^ = .06. A closer examination of univariate effects indicated that emotion dysregulation decreased over time, regardless of group membership or gender. No difference was observed in depressive symptoms over time. The main effect for group was also significant, Wilks’ λ *=* .60, *F* (4, 218) = 15.79, *p* = .00, ηp^2^ = .23. A closer examination of the univariate effects indicated that as expected, the comparison group reported significantly less emotion dysregulation and depressive symptoms when compared to the NSSI Stop and NSSI Maintain groups. Moreover, the NSSI Maintain group and NSSI Stop group also differed, with the NSSI Maintain group reporting significantly more emotion dysregulation difficulties and depressive symptoms. The main effect for gender was not statistically significant, Wilks’ λ = .98, *F*(2, 109) = 1.43, *p =* n.s., ηp^2^ = .03, indicating that adolescent males and females did not differ significantly in emotion regulation or depressive symptoms, regardless of their NSSI status.

No interaction effect was found between NSSI status and gender, Wilks’ λ *= .*95*, F*(4, 218) = 1.29, *p =* n.s., ηp^2^ = .02, or between time and gender, Wilks’ λ *= .*99*, F*(2, 109) = .83, *p =* n.s., ηp^2^ = .02, or between NSSI status and time, Wilks’ λ *= .*93*, F*(4, 218) = 2.05, *p =* n.s., ηp^2^ = .04. However, the triple interaction between NSSI status, gender, and time was significant, Wilks’ λ = .91, *F*(4, 218) = 2.58, *p* = .04, ηp^2^ = .05. A closer examination of univariate effects indicated that emotion regulation, and not depression, significantly changed across group and gender as a function of time, Wilks’ λ *=* .07*, F*(2, 110) = 4.09, *p =* .02, ηp^2^ = .07. Specifically, males in the NSSI Stop group reported significantly less emotion regulation difficulties at T2 when compared to the females in the NSSI Stop group. Refer to Table 4 for a detailed presentation of the univariate effects of group and gender on emotion dysregulation and depression across time.

**Table 4:** Univariate Effects of Group and Gender on DERS and BDI Across Time

Dependent Variables	*df*	*F*	ηp^2^	Observed Power	*p*
Group Membership					
DERS	2	12.84	.19	1.0	.00**
BDI	2	35.19	.39	1.0	.00**
Gender					
DERS	1	0.73	.01	.14	.40
BDI	1	2.88	.03	.39	.09
Time					
DERS	1	6.10	.05	.69	.02*
BDI	1	1.85	.02	.27	.18
Group X Time					
DERS	2	0.33	.01	.10	.72
BDI	2	4.16	.07	.72	.02*
Time X Gender					
DERS	1	0.28	.00	.08	.60
BDI	1	1.15	.01	.19	.29
Time X Group X Gender					
DERS	2	4.09	.07	.72	.02*
BDI	2	1.05	.02	.23	.35

* = *p* < .01; ** = *p* < .001

## Discussion

The current study applied objectification theory [25] as a theoretical lens to explore associations between objectified body consciousness, depressive symptoms, emotion dysregulation, gender, and NSSI engagement longitudinally among a community sample of early adolescents. The research objectives were twofold. The first objective was to investigate group and gender differences among dimensions of body objectification (i.e., body shame, body surveillance, appearance control beliefs) among three groups of interest (i.e., NSSI Maintain group, NSSI Stop group, comparison group) over time. The second objective was to examine changes in depression and emotion dysregulation across groups and gender over time. The present findings offer the first study to examine temporal associations between body objectification, depression, emotion dysregulation, and gender among an early adolescent sample of males and females.

### Body objectification

A significant group by time interaction indicated that both NSSI groups reported greater body shame and body surveillance when compared to the comparison group, however, at T2, the NSSI Maintain group reported significantly more body surveillance than both the NSSI Stop group and the comparison group. As previously summarized, a growing body of literature supports an association between body objectification, body image, and NSSI engagement among adolescents and young adults [18, 23, 24]. However, as the first longitudinal study to investigate body objectification and NSSI, findings from the present study offer an understanding of how body objectification relates to NSSI engagement over time. More specifically, our findings suggest that body surveillance, which refers to appearance monitoring and adopting an outsider’s view of the self, represents a critical variable associated with differentiating adolescence who are presently engaging in the behaviour and adolescents who have recently stopped. Our findings suggest that body surveillance may represent an important risk factor associated with the continuation of the behaviour over time, and possibly, a more severe presentation of the behaviour. Additionally, given previous findings [28] which suggest that negative body image and low self-esteem are comparable among adolescents who engaged in NSSI, and adolescents who engaged in NSSI and report a history of suicidality, it would be of interest to further evaluate body surveillance between varying presentations of self-harming behaviours (i.e., no history of self-harming behaviours, NSSI only, and NSSI with a history of suicidality) among both clinical and community samples.

In the present study, youth were transitioning to adolescence, a time where the ability to cognitively engage in body objectification also typically begins to emerge [30, 31]. Previous literature has focused on examining body objectification among young adults and later adolescents [18, 24]. Thus, findings from the current study also validate the presence of body objectification as a risk factor associated with NSSI engagement among an earlier adolescent age group than previously examined (i.e., 11 to 12 years of age). Given that adolescence is a developmental period that confers particular risk for NSSI, as it represents a time that NSSI behaviour typically manifests [11, 12], understanding core risk factors associated with the cessation and/or maintencance of the behaviour are valuable in partially explaining why the developmental period plays such a crucial role in the etiology of NSSI. Additional longitudinal studies that incorporate an NSSI onset group (i.e., adolescents who begin engaging in NSSI over the course of the study) are needed to parcel out the nature of the relationship between NSSI and the body. Specifically, this would clarify whether negative body experiences are a precipitating risk factor or in fact an eventual consequence of continued engagement in NSSI behaviour. A recent study [42] reported that self-esteem and self-efficacy were significant predictors of NSSI onset among a community sample of adolescents. Furthermore, self-esteem [28] is a critical risk factor in differentiating adolescents who engaged in NSSI and those who do not. Additionally, a substantial body of literature suggests that childhood maltreatment negatively influences how an individual views oneself [43]. As childhood maltreatment represents a robust risk factor associated with NSSI [44] future studies would be well served to explore maltreatment as a precipitating factor associated with the development of a negative relationship with the body, and eventual NSSI engagement. Given these findings, and the large degree of overlap between body objectification and self-esteem, future studies would benefit from examining the role of body objectification in conjunction with other intrapersonal variables (i.e., self-esteem, self-efficacy, emotion dysregulation) and risk factors (i.e., childhood maltreatment) to determine which variables instigate the onset of NSSI and whether the same factors dictate continuation over time.

Results indicated that the comparison group reported an increase in body shame and body surveillance over time, as well as a decrease in appearance control beliefs. These findings are congruent with previous research, which suggests that both adolescent girls and boys report significant concerns over their bodies during this time [30, 32]. As previously mentioned, body objectification typically emerges at this time, and early adolescence represents a particularly critical period for body objectification due to numerous normative developmental changes, including pubertal onset, emerging sexuality, identify formation, and gender role intensifications [30]. Taken together, these findings offer partial explanation regarding the comparison groups elevated body objectification, and highlight the importance of acknowledging body objectification as a concern among typically functioning early adolescents.

### Emotion dysregulation and depression

The second objective was to examine changes in depression and emotion dysregulation across groups and gender. Findings indicated that the non-NSSI group reported significantly less emotion dysregulation and depressive symptoms when compared to the NSSI Stop and NSSI Maintain groups over time. Moreover, the NSSI Maintain group and NSSI Stop group also differed, with the NSSI Maintain group reporting significantly more emotion dysregulation difficulties and depressive symptoms at T2. It is likely that adolescents with a greater capacity to regulate their emotions and less emotional distress may be less likely to continue engaging in NSSI, resulting in the cessation of the behaviour. However, in the absence of such protective factors, these adolescents may be more likely to continue with NSSI behaviours, with potential increases in frequency and severity.

When these findings are taken in conjunction with the elevated body surveillance and body shame reported by the NSSI Maintain group, and previous findings [24], it appears that adolescents who reported an objectified body consciousness are likely to continue engaging in NSSI behaviour when confronted with overwhelming emotional distress and an inability to regulate emotions [21, 29]. These results validate the objectification theory, as a combination of emotionally based risk factors and objectified body consciousness (specifically body surveillance) appear to be related to the maintenance of NSSI behaviour among early adolescents.

### Limitations

Although the longitudinal design and low attrition rate represent strengths of the study, results should be interpreted in light of the limitations of the study. To begin, the community-based sample was comprised of a homogenous group of typically functioning adolescents. It remains unclear how these findings would generalize to a more diverse group of adolescents with different pathologies, or to an inpatient or outpatient clinical sample. A second limitation concerns the use of self-report measures. Specifically, when informed consent was being explained to participants, they were notified that their school mental health professional would be contacted in the event their responses indicated a risk of self-harm or harm to others. Therefore, it is possible that a portion of adolescents may have censored their answers and chose not to fully disclose certain stress and coping strategies when completing certain measures. Furthermore, although the longitudinal nature of the study allowed for the examination of change over a 12-month time period, the two data points only provided a brief depiction regarding developmental change. Future studies would benefit from using growth curve analysis, as this would provide insight regarding both individual and group temporal growth trajectories; however, growth models typically require at least three time points per individual [45]. This would allow for the examination of change at both an individual and group level, across a broader time span of development in relation to NSSI. Furthermore, given the various alternative (e.g., “Goth” or “Emo”) subcultures within the NSSI population [46], future studies would benefit from further exploring the role of body objectification across different subcultures within the NSSI population. Another limitation concerns how emotion dysregulation was measured, as it consisted of only three items, which limited the range of information assessed. Given the well-documented association between emotion dysregulation and NSSI engagement and the results from the current study, future studies would benefit from including a more robust measure of emotion regulation. This would also provide for an in-depth understanding of how changes in specific areas of emotion dysregulation relate to body-oriented variables, depression, and the course of NSSI. Finally, future studies should include additional measures of body-related variables (e.g., interoceptive awareness, dissociation, body esteem, self-esteem, and self-concept) in addition to dimensions of self-objectification, to obtain a more comprehensive understanding of the nature of the relationship between the body and NSSI engagement.

### Clinical implications and summary

Although the present results are preliminary and in need of replication, the present study findings are critical to the assessment of NSSI and directions for intervention. Findings from the current study, as well as from a growing body of research, highlight the need to include body-related variables into both risk assessment as well as treatment approaches for youth who engage in NSSI. Furthermore, with respect to risk assessment, it appears that body-related concerns are useful indicators of both a history of NSSI and current engagement in NSSI during early adolescence. Evaluation of body experiences, emotion regulation, and depressive symptoms, may assist clinicians in identifying youth at-risk for NSSI engagement, or youth who have a history of NSSI. Body and self-oriented variables, including self-concept, self-esteem, body image, and self-objectification, represent related and malleable risk factors that are subject to influence and change over the course of development. This alone has important clinical implications, as decreases in negative body image may represent one mechanism through which to achieve therapeutic change [47, 48]. Thus, treatment approaches should focus on taking a strengths based approach to repair negative body image, including fostering positive self-esteem and positive body image development to improve one’s relationship with their body [49]. For example, treatments that incorporate mindfulness training (e.g., dialectical behavioural therapy) or body image work [50] may be more effective at reducing NSSI, because they focus on improving body awareness, body acceptance, and body integrity [50]. Moreover, acknowledging the role of objectification and the broader sociocultural context in therapeutic sessions has strong implications for prevention and intervention efforts. Clinicians should focus on developing client insight regarding how socio-cultural context contributes to body objectification processes and body dissatisfactions, and the implications that this has for their self-esteem and overall self-concept. One method to do so is by priming youth and parents to be critical consumers of media [51]. Additionally, open discussions with the family as a unit could offer potentially valuable avenues in understanding the influence of objectified body consciousness on parent-adolescent relations.

This study represents a contribution to the current literature on body objectification and NSSI as it identifies body surveillance, as a critical factor associated with the maintenance of NSSI among an early adolescent community sample. Despite study limitations, the current findings provide direct support for the role of body objectification, emotion dysregulation, and emotional distress as factors associated with NSSI engagement among young adolescents. It appears that body surveillance may be a particularly salient mechanism to consider both in understanding and treating NSSI. Although further replication and continued investigation is needed, it appears that inclusion of body surveillance, as a risk factor will result in a more comprehensive etiological model of NSSI risk.
